# Mediterranean Journal of Rheumatology March 2020 Highlights

**DOI:** 10.31138/mjr.31.1.1

**Published:** 2020-03-31

**Authors:** Katerina Chatzidionysiou

**Affiliations:** 1 Department of Propaedeutic and Internal Medicine, “Laikon” Hospital, Medical School, National and Kapodistrian University of Athens, Athens, Greece

We are witnessing a global pandemic that has hitherto killed more than 30,000 people worldwide, and the number of confirmed cases has reached to date almost 660,000. The COVID-19 pandemic has imposed unprecedented changes in all aspects of our lives and in our societies. We have a duty, as a scientific medical society, not only to be at the forefront of patient care, but also to provide information to our patients and to create evidence that will inform our clinical decision-making now and in the future.

The official statement of the Greek Rheumatology Society and Professional Association of Rheumatologists (EREEPERE) regarding COVID-19 infection is published in the March 2020 issue of the MJR.^2^ This proposal tackles important aspects of COVID-19 in relation to rheumatic disease, such as, whether patients with rheumatic diseases are a ‘’vulnerable group’’, what is the risk of a serious outcome of the infection for patients receiving anti-rheumatic treatment, and what should patients with rheumatic disease who receive anti-rheumatic treatment do. The document provides important information and useful links for rheumatologists and patients and will be updated as more data becomes available.

An interesting article by Bogdanos et al.^17^ discusses whether hydroxychloroquine, a drug that rheumatologists and their patients know very well and use routinely for decades, could be part of our armamentarium against COVID-19 pneumonia. Clinical trials on this are currently ongoing and may well provide a definitive answer soon.

Despite these difficult times, the focus on COVID-19 and the increased clinical load, we still need to continue with our clinical and research activities in our field of rheumatology. The March 2020 issue of MJR is rich with clinical and research material from various groups from 6 different countries.

The need for real-life evidence for better understanding of disease prevalence, phenotype, treatment modalities and outcomes is increasing. In the March 2020 issue of the MJR, the initiatives for two large registers sponsored by ERE-EPERE are presented. The first one is a contemporary, web-based and easy-to-use patient registry specifically for patients with antineutrophil cytoplasmic antibody (ANCA) associated vasculitides (AAV).^12^ The purpose of the project is to increase our understanding of AAV in our country, with a future prospect of contributing to and sharing data with other international registries. The second one is a pilot study of an electronic registry for monitoring Systemic Lupus Erythematosus (SLE) patients who are treated with novel/biologic therapies.^13^ The therapeutic armamentarium in SLE is expanding with the introduction of novel biologic and small-molecule agents. Registry-based studies with high external validity and long-term follow up of patients are needed. Moreover, data from registries can be used to identify disease phenotypes that best respond to biologic agents, and to correlate clinical response with parameters such as co-administered therapies and comorbidities. With regard to this, Kourti et al.^8^ provide insights into the regulation of microRNAs, namely miR-21 and miR-22 in SLE, and the preliminary results of this research indicate a potential role of these molecules in the assessment of disease severity.

Peripheral immune-mediated polyneuropathies (IMPN) are a diverse group of rare neurological illnesses characterized by nerve damage. In an interesting review by Dr Roggenbuck et al.^4^, the potential implication on the stratification of patients and their treatment response of the progress in testing for autoantibodies against glycolipids or paranodal/nodal molecules is analysed.

In another interesting review by Fenton et al.,^5^ interventions that may help reduce sedentary behaviour and promote physical activity in patients with rheumatoid arthritis are discussed in depth. Lifestyle modification is now recognised as a major part of care in most rheumatic and musculoskeletal diseases, and several of the concepts described here may be generic and transferable to other disease entities. A number of very interesting case reports are included in this issue. In a case-report by Dr Partalidou et al.,^10^ a rare case of rhombencephalitis in a patient with known neuro-Behcet’s disease is described. It is known that Anti-melanoma differentiation associated gene 5 (anti-MDA5) autoantibodies are associated with amyopathic dermatomyositis and in particular with rapidly progressive interstitial lung disease. Dr. Zohar et al.^9^ discuss the prognostic value of these autoantibodies and ferritin levels in the outcome. On the top of the case reports, a clinical image communication addresses the differences between diffuse idiopathic skeletal hyperostosis (DISH) and spondyloarthropathies on radiological grounds; a very common dilemma in daily clinical practice.^11^

In a very interesting original article, Dr Gkoutzourelas et al.^7^ present a plethora of novel microbial mimics, which share remarkable amino acid similarities with the respective autoantigenic epitopes, using a comprehensive bioinformatic analysis. Examination of the role of miR-21 and miR-210 in patients with SLE is the aim of an original study by Dr Kourti et al.^8^, and in this issue some preliminary results of this study are reported.

Osteoporosis is known as a silent disease and is commonly underestimated by rheumatologists. In this issue, MJR hosts a large epidemiological study on osteoporosis recruited 731 active elderly Majorcan individuals.^6^ The authors found a higher than expected prevalence of osteoporosis despite the favourable characteristics of their population (physically active persons, habitants in sun-exposed area). Age and female sex were –normally-determinants of osteoporosis, suggesting that intelligent surveillance should be implemented in elderly population across Mediterranean countries.

In a commentary, Prof. Moutsopoulos describes the current situation of Rheumatology in Greece, strengths and areas of improvement, with special focus on education of future rheumatologists and research production.^3^

We take the opportunity to acknowledge the tremendous contributions to the field of autoimmunity of Prof Ian Reay Mackay who passed away recently, in an obituary by Prof. Bogdanos.^16^

Last, but certainly not least, we would like to share with you some good news. The *MJR* has recently achieved a major milestone for any “young” scholarly journal: it has been accepted for indexing by PubMed and archiving by PubMed Central (PMC). The importance of this for any journal, including MJR, is discussed in the Editorial by Gasparyan and Kitas.^1^ This is a major achievement that could not have been realised without the dedicated effort, hard work and support of the editorial team, reviewers, authors, and, of course, the vision and continuing support of the Greek Rheumatology Society and Professional Association of Rheumatologists.

We would like to wish you all physical and mental health, during these challenging and unprecedented times. We need to remain optimistic, physically and mentally strong, as this will be key to our psychological resilience in overcoming this challenge united and in order to be able to provide our help and support to those who need us the most.
